# Sociocultural influences on newborn health in the first 6 weeks of life: qualitative study in a fishing village in Karachi, Pakistan

**DOI:** 10.1186/1471-2393-14-232

**Published:** 2014-07-16

**Authors:** Shahirose Premji, Shaneela Khowaja, Salima Meherali, Rachelle Forgeron

**Affiliations:** 1 Faculty of Nursing, University of Calgary, 2500 University Drive NW, Calgary, Alberta T2N 1N4, Canada; 2 Faculty of Medicine, University of Calgary, TRW Building, 3rd Floor, 3280 Hospital Drive NW, Calgary, Alberta T2N 4Z6, Canada; 3 School of Nursing and Midwifery, Aga Khan University, Stadium Road, P.O. Box 3500, Karachi 74800, Pakistan; 4 Faculty of Nursing, University of Alberta, Level 3, Edmonton Clinic Health Academy, 11405 87 Avenue, Edmonton, Alberta T6G ICP, Canada

**Keywords:** Caregiving environment, Community health care, Infant, Newborn, Qualitative, Pakistan

## Abstract

**Background:**

Given regional variability and minimal improvement in infant mortality rates in Pakistan, this study aimed to explicate sociocultural influences impacting mothers’ efforts to maintain or improve newborn health.

**Methods:**

We used a qualitative phenomenological approach. A total of 10 mothers and 8 fathers from a fishing village in Karachi, Pakistan were purposefully sampled and interviewed individually. A focus group was undertaken with four grandmothers (primary decision makers). Transcripts were independently reviewed using interpretive thematic analysis.

**Results:**

A multigenerational approach was used in infant care, but mothers did not have a voice in decision-making. Parents connected breast milk to infant health, and crying was used as cue to initiate feeding. Participants perceived that newborns required early supplementation, given poor milk supply and to improve health. There were tensions between traditional (i.e., home) remedies and current medical practices. Equal importance was given to sons and daughters.

**Conclusion:**

Findings suggest that social and cultural influences within families and the community must be considered in developing interventions to improve newborn health. Introducing non-breast milk substances into newborn diets may reduce the duration of exclusive or partial breastfeeding and increase risks to infant health.

## Background

Over three million babies die in the first 4 weeks of life each year (i.e., neonatal deaths), 99% of whom lived in the developing world [1]. Pakistan ranks third highest in the world for the rate of neonatal deaths per year [2], and though Pakistan has implemented national programs for maternal, newborn and child health, childhood death rates have been declining faster than neonatal deaths [3,4]. There is also significant variability in rates within and between provinces [5]. For example, the risk of neonatal death is higher in rural (55 per 1000 live births) compared to urban areas (48 per 1000 live births) [6]. Although progress has been made to meet Millennium Development Goals 4 (i.e., reduce child mortality) established during the Millennium Summit of the United Nations in 2000 [3]; significant work remains to reduce neonatal deaths across the country [3].

Research on predictors of neonatal death in developing nations has predominantly focused on medical, socioeconomic, and demographic factors e.g., [7-10]. Less is known, however, about situational facilitators or barriers to mothers’ efforts to maintain or improve newborn health. Programs for newborn survival in Pakistan have not improved neonatal survival despite investment of funding, and policy and program changes given diverse practices, including the social norms dissuading mothers from timely care seeking [5]. Individual conceptions of health and illness, and health practices are influenced by cultural beliefs, values, expectations, and sociocultural factors (e.g., immigration, acculturation) [11]. Moreover, the physical environment and larger social structures (culture, organization, and policy) [12], such as extended or joint family systems that are commonplace in South Asia, are especially important to the health of the poorest and most vulnerable members of society [13].

The purpose of this study was to understand the process through which sociocultural factors shape how mothers care for and decide to seek health care for their newborns in the first 6 weeks of life. Rather than viewing individual women as wholly responsible for the health care decisions for their infant, this study elucidated behaviors that were dictated by their cultural beliefs, expectations, gender roles, and hierarchical relations. Through systematic analysis of these sociocultural influences, we hoped to identify interactions that go beyond the biomedical model of health care and consider complex contextual issues (e.g., culture, gender) that influence the mothers’ efforts to maintain or improve newborn health.

This qualitative study addressed two research questions:

1. How do Pakistani women residing in a fishing village participate in the decision-making process in caring for and seeking health care for their newborn?

2. What are the key factors that influence this decision-making process?

## Methods

A descriptive qualitative research design using a phenomenological approach illuminated the mothers’ experiences to maintain health or improve the health outcome of her newborn and the meaning she assigned to these experiences. In the phenomenological method, extensive and prolonged engagement with mothers uncovers the “essence” of their experiences by recognizing patterns and relationships of meaning [14]. The interview was the place where through conversation the phenomenon was explored in an intentional way, while remaining open to directions opened up by the conversation [15]. To understand the process through which social life and social relations have organized women’s lives and shaped how they care for and seek health care for their newborns, the aim of the conversations was to identify the meaning women gave to the patterns created in their daily lives.

Purposive sampling was employed to recruit mothers of infants less than 6 weeks of age, their husbands and their family’s primary decision-makers from Ibrahim Haidery, a fishing settlement situated in Bin Qasim town, Pakistan. The community of nearly 60,000 Sindhi-, Baloch-, and Bengali-speaking people has a government hospital, private hospital, trust clinic, and many private clinics with general practitioners. The neonatal mortality rate in this community is 21 per 1000 live births. Although the risk of neonatal mortality in Ibrahim Haidery was lower than national rates, it was selected as the study setting as the Aga Khan University, Department of Paediatrics and Child Health has a longstanding relationship with this community.

Women were eligible to participate in the study if they (a) had an uncomplicated normal delivery and live baby birth in the month the study commenced, (b) were primigravida or multigravida, (c) had a singleton or twin pregnancy, and (d) were from a nuclear or an extended family who delivered in the month the study commenced. We deliberately attempted to select a heterogeneous sample based on the age and socioeconomic status of the mothers, and the age and sex of the infant, which would provide significant input into how mothers care for and seek health care for their newborn in the first 6 weeks of life (see Table 1 for participant and infant characteristics). Participants were excluded if their infant was older than 6 weeks of age. Drawing from a socio-ecological model, fathers and primary decision-makers were included as participants to develop a more complete understanding of the physical and social environment (e.g., individual, family, cultural) that could facilitate or hinder the mother’s efforts to maintain health or improve the health outcome of her infants [12]. A well-known resident of Bin Qasim Town and a trained traditional birth attendant, who maintained pregnancy and birth records, assisted in identifying and recruiting participants by meeting eligible mothers in their home. Field workers used a standard script to share study information to eligible participants who indicated a willingness to participate. Permission was first obtained from the mother, who identified the primary support person assisting in newborn care (i.e., grandmothers). Permission was then obtained from the husband and grandmothers. At least one household member had to be willing to participate before field workers subsequently negotiated a mutually-convenient date and time for either an interview or focus group discussion. Each family household was asked to complete a demographic questionnaire; this was completed individually by the mother or as a collective (i.e., in the presence of the father and grandmother).

**Table 1:** Characteristics of mothers, their husbands, and their infants

**Characteristic**	**Frequency (%)**
**General**	
Family structure (n = 10)	
Nuclear	5 (50%)
Extended	5 (50%)
No. members in household (range)	3 – 14 people
Annual family income after taxes (n = 10)	
Rs $10,000 to 49,999 (US $110-$550)	2 (20%)
≥ Rs $50,000 (US $550)	8 (80%)
Mother^*^	
Age, years (n = 8)	
Range (median)	26 to 35 (31)
Marital Status (n = 10)	
Married	10 (100%)
Language (n = 10)	
Sindhi	10 (100%)
Urdu	9 (90%)
Education (n = 9)	
Not educated	5 (50%)
Below grade 5	2 (20%)
Between grade 5 and 10	2 (20%)
Employment (n = 2)	
House-hold chores	1 (50%)
Part-time work	1 (50%)
Parity (n = 10)	
Primiparous	3 (30%)
Multiparous	7 (70%)
Primary responsibility of child (n = 10)	
Mother-in-law	3 (30%)
Mother	7 (80%)
Father^*^^	
Age, years (n = 9)	
Range (median)	25 to 37 (32)
Language (n = 10)	
Sindhi	10 (100%)
Urdu	7 (70%)
Baloch	1 (10%)
Education (n = 10)	
Not educated	5 (50%)
Below grade 5	3 (30%)
Between grade 5 and 10	1 (10%)
Graduate education	1 (10%)
Employment (n = 10)	
Full-time	6 (60%)
Part-time	4 (40%)
Occupation (n = 7)	
Fisherman	5 (50%)
Hotel job	1 (10%)
Government Employee	1 (10%)
Child^*^	
Sex (n = 9)	
Male	3 (33%)
Female	6 (67%)
Birth weight (n = 2)	
Low birth weight	1 (50%)
Age at time of interview (n = 9)	
≤ 1 week	4 (40%)
1-2 weeks	4 (40%)
2-3 weeks	2 (20%)
Breastfeeding at the time of contact	10 (100%)
Illness since birth	
Yes (fever, jaundice, blue at birth)	3 (30%)
No	7 (70%)
Hospitalized since birth	
Yes (2 hours for “water in abdomen”)	1 (10%)
No	9 (90%)
No. of siblings	
Range	3 to 7
Females	23
Males	12

*Number less than 10 reflect missing data.

^The mother individually or in consultation with the family (i.e., father/grandmother) completed one demographic questionnaire for the household. The mother, father, and primary decision-maker were from the same household.

### Data collection

**Figure 1:**
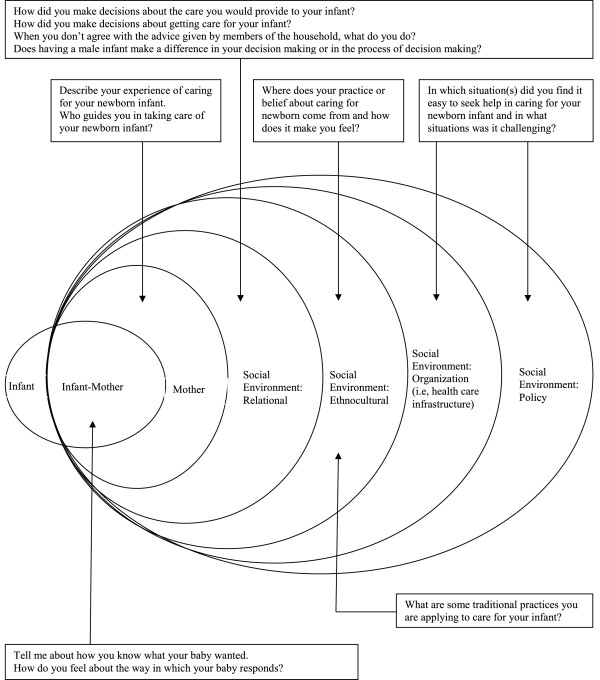
Theoretical framework: Interview guide for individual interviews.

**Figure 2:**
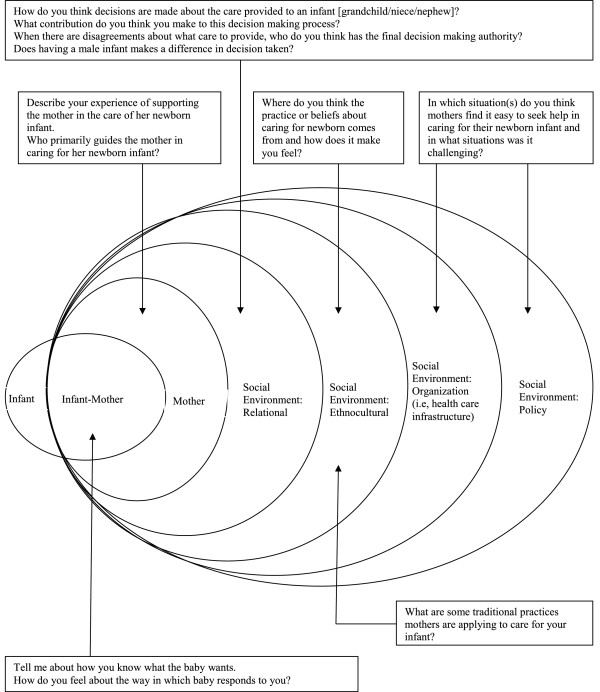
Theoretical framework: Interview guide for focus groups.

Between April and August 2010, semi-structured interviews were conducted with 10 mothers and 8 fathers in their homes. A total of 4 grandmothers participated in a focus group at a community center. The focus group approach was intended to promote participation as it engaged the grandmothers in natural conversation. There were 2 female interviewers, 1 of whom served as primary interviewer for all interviews. Fathers were interviewed by the male field worker. The hour-long, individual interviews and focus group were conducted in the local language (i.e., Sindhi) and audiotaped with the permission of the participants. A socio-ecological model [12] guided questioning, permitting a comprehensive understanding of the physical and social environments (e.g., individual, family, cultural, organization, or policy) that impact mothers’ efforts to maintain or improve the health outcome of their infants (see Figures 1 and 2). Interviews began with a general request to describe the experience of caring for the newborn infant and how decisions about care provided to the newborn are made. Field notes were made after each interview. Audiotapes were transcribed in the original language, and then translated into English prior to analysis.

### Data analysis

Transcripts and field notes were analyzed using Colaizzi’s phenomenology method [16]. Step 1: As data were obtained, interviews were transcribed and rechecked against the audiotapes, then translated to English and verified. Step 2: The data was reviewed several times by 2 research team members (SP, RF) to become familiar with the content. Each transcript was read thoroughly line-by-line, identifying significant statements related to circumstances that impacted mothers’ efforts or actions to improve maintain or improve newborn health by marking or commenting in the margins of the transcript. Step 3: Meanings were formulated in relation to each statement. The 2 research team members then met to discuss the statements, meanings, and aggregated meanings were organized into clusters or themes. Disagreements were resolved by returning to the original audiotape and English text and coming to consensus. Step 4: A composite summary of statements, interpretation of meaning, and a saturated description of themes were written (see Results).

### Rigor and trustworthiness

We identified women who were willing to be trained and work in the field as a paid worker, and who resided at the study site and were able to read and write Urdu (the lingua franca). These women were trained in administering study questionnaires (e.g., socio-demographics), undertaking guided interviews, and providing counselling sessions to mothers who were anxious or depressed to help them cope. To ensure continuity across interviews, 3 bilingual field workers (spoke Sindhi and Urdu) were also trained in undertaking semi-structured interviews. One author (SK) speaks and writes Sindhi, and another (SP) speaks Urdu and Katchi (a dialect of Sindhi), hence they were able to coach the field workers during training interviews. The research team also accompanied the field workers during the initial 3 or 4 interviews. All interviews were included in the analysis. To confirm the validity of the themes, 2 research team members independently reviewed and analyzed data. During this process, transcripts were reviewed again to confirm themes and ensure the meanings are informed by the data. The reviewers engaged in conversation to resolve differences and clarify interpretations and linking excerpts from the participants’ raw data [17].

### Ethical considerations

Prior to undertaking the study, verbal and written permission were secured from the Nazim (leader) of Bin Qasim Town and the Department of Paediatrics and Child Health Department, Aga Khan University. The Aga Khan University Ethical Review Committee approved the study (1502-SON-ERC-2010). Both verbal and written consent were obtained from the participants prior to each interview and focus group.

## Results

Since qualitative research is a situated activity [18], we wish to provide some salient observations about engaging the participants. Semi-structured interviews intend to allow interviewees to provide additional information where needed, while ensuring broad themes and ideas are explored [19]. This technique proved more successful with fathers, who often explored their answers in detail. The women, especially mothers, often answered with just a few words. Mothers frequently struggled answering broad questions, sometimes needing questions to be re-phrased and more specific. Fathers and grandmothers often provided more elaborate answers including information about the context in which these families live (e.g., financial status, working conditions). While it was challenging to encourage mothers to express themselves, adequate data was gathered for thematic analysis. Themes that emerged are described below in relation to each research question.

### Participation in decision-making around newborn care

#### All together we do it

Mothers are the primary care givers of infants, but all participants explained that families work closely together to raise an infant. Mother 3 stated, *“I am there, my mother-in-law is there, aunt [name] is there. All together we do it.”* Each family member had specific roles and responsibilities. Mothers were responsible for the basic care needs of the infant such as *“tak[ing] care of her, clean[ing] her, feed[ing] her milk”* (Mother 3). Grandmother 2 described her role as *“washing clothes, massaging the baby, bathing, and making him sleep, all these things”*. Fathers provided situational support:

*I help my wife a lot…When [the child] cries [at night] a lot, then I take her from her mother and I take her outside for a round.* (Father 3)

#### Nobody is born learned

One reason families work together to raise their infant is that parenting competencies are built and taught within the families.

*My parents have taught me…Nobody is born learned…we will learn these worldly matters.* (Father 5)

Furthermore, participants had confidence in knowledge transferred from one generation to the next, as they had survived and grown because of this care. Father 7 spoke of the trust he had in his mother’s caring skills:

Our mother taught us this that how to take care of child that how to feed child…When mother took care of us and made us grown so today also she knows that how to take care of child.

#### Feeding mother’s milk

All babies were being breast fed, the primary role of mothers: *“I feed him…I only breast feed the child”* (Mother 1). Breast milk was considered *“more nutritious,”* and *“important for the child; nothing is better than that”*. Consequently, mothers were encouraged to breast feed. Crying was identified as a hunger cue: *“when she cries I come to know that she is [hungry]”* (Mother 2). Persistent crying, on the hand, was a sign of illness and prompted parents to seek guidance to problem solve next steps of care:

*Yes, understand that [baby] cries too much then I goes to tell [the grandmother] that she is crying so she looks after that what has happened then she tells me that feed her or give medicine.* (Mother 3)

#### Hunger not met by breast milk

Although some mothers indicated that they only breast fed their baby, upon further probing, mothers disclosed use of home remedies and supplementation:

*Breast milk was not secreted yet, then my mother in law said that give him ‘sulemnai chaye’ for three to four days, so that milk gets secreted by then.* (Mother 1)

Supplementing also occurred during other critical time, generally identified as after 40 days, to promote growth of the child. Mother 2 explained, *“when [small baby girl] will be forty days old, then my mother-in-law used to say that now her hunger will not meet by this milk, give her something else to feed”*. Fathers shared their perspectives on hunger and supplementation:

*Mother’s milk is like water; as we adults eat food, so our hunger gets satisfied and the water quenches our thirst, so we discuss with each other that the child’s hunger is not satisfied with mother’s milk and we will have to do something else for this. Then we bring those foods that the doctors prescribes. We bring cerelac.* (Father 3)

### Newborn illness and decision-making processes to seek health care

#### Consulting the elders

Extended family members, especially paternal grandmothers, were a significant source of support for parents in problem solving the care needs of their infant:

*Our elders know more than us, they have raised us; so we will consult them. If they would say that ‘let’s take the child to the doctor and he will be fine,’ or if they would say that ‘he has this problem, manage it like this then he will be alright;’ so we will do that only.* (Father 4)

Elders outside the family were also consulted about treating illness: “*after asking from five to six ladies* (about old remedies) *then I do it. Otherwise I don’t do it”* (Father 8). Fathers also acknowledged other supportive family members. Father 8 indicated that his sister-in-law taught him to *“cover the baby in cloth or to tie or understand to bath him/her or to massage”.*

#### Taking permission and doing only as the elders say

Although a source of support, paradoxically, paternal grandmothers also contributed to mothers’ psychological distress. Initially, most mothers conveyed independent decision-making for their infant or children, but it became clear with further probing that both mothers and fathers needed some form of permission in order to make decisions for the health of their infant:

*No, I [make a decision] after asking from elders. I have mother-in-law… I ask her that what I should do, if she forbade me for something, I don’t do that when whatever she says I do accordingly.* (Mother 3)

Although Grandmother 1 indicated, *“mostly this decision lies with the parents”* when seeking clarification, she indicated *“my decision will followed”.*

#### Keeping quiet

Advice from elders is pivotal to the decision-making process. However, mothers’ voices are often not heard before a decision is finalized. Occasionally, this may be because mothers feel confident in elders’ decisions. Other times, mothers expressed an inability to speak out against the decisions of their family members: *“Yes, I do feel sad [about the decision] but then I keep quiet”* (Mother 1). Several fathers expressed similar sentiments: *“I don’t do anything. I just go and sit down silently. I will see that really it is appropriate”* (Father 5). Other fathers offered their perspectives, particularly when the child was not getting well*: “I will say that this remedy is not right. I will say that I have seen or heard of these two three remedies”* (Father 3). For some, the desire to do everything for the child meant going against the elders:

*If even [our elders] won’t be able to handle it, then we will consult a doctor, so that he checks him and tells us that what the problem with the child is. God has given huge power to the doctor, so they treat the patient well. Then life is in the hands of God.* (Father 4)

#### Problem solving and being prepared

Grandmothers based health-seeking behaviors on certain cues. Father 3 explained:

*The [mother-in-law] will lick the child’s hand and if she will find it salty, then she will understand that the child has stomachache. Then we will take [the child] to the woman who applies ‘janrrio ungrrion’* (twin fingers)*. It is very effective.*

One grandmother indicated that it is difficult to determine the health needs of the infant prior to the 40 days:

*We do not know about the child’s pain till he gets 40 days old. Then he will touch his ear again and again, then we will say that he has earache.* (Grandmother 2)

Being prepared was important since *“illness is unpredictable and the child can fall ill at any time, therefore we make arrangements for this beforehand”* (Father 3). This included buying and keeping medicine at home *and “do these remedies, and if something serious happens then we take the child for the ‘parainnr’ (dum)”* (Grandmother 3).

#### Tension between home remedies and medicine

Grandmothers spoke of changes to illness treatment:

*“Previously, the children were different. Elder women used to sell home remedies. Nowadays, even if something minor happens, [mothers] take the child to the doctor. Previously, they used home remedies*”. (Grandmother 4)

Father 7 validated this shift in perspective stating “*Among us doctor is more authoritarian [then the elders]. Whatever doctor prescribes they treat child accordingly. Except this we don’t do anything”.* As the medical community becomes more involved in the community, the information family members received is not always consistent with the community’s cultural practices. There is conflict between the ways in which the community cares for infants and the recommendations from the medical professionals.

*Now the children of new generation like to have new and prepared products…. they use those things and they say that ‘we do not trust those [old] methods.’* (Grandmother 2)

#### Doing everything for the child

Regardless of the approach to care (i.e., home remedies or medicine), the ultimate aim was *“so that [the] child gets well…When the child get alright, there is no other moment of happiness like that”* (Father 4). All participants were deeply affected by the health of their infant, compelling them to do anything for their child. Mother 2 stated*, “For kids, person can do everything. I will not wait for others”.* Additionally, fathers verbalized a deep love for their infant, acting as a motivating factor:

*It seems as if my child is my heart, he is my soul and a part of mine. The way a father cares for his child, I care for him that way only. The child is a part of the parent. Anybody who cares for his child does everything he can for the child.* (Father 9)

All participants resolved at some point that the outcome for the child was *“dependent on God”.*

### Factors influencing decision-making

#### Poverty

Despite the deep love for their child, fathers also expressed difficulties in caring for their families because of their financial status:

*I am a poor man, whatever I earn and all the efforts that I can do, I will do those for my child, and I will not let my child suffer for anything.* (Father 9)

Fathers spoke about providing for their infant, *“the child should not feel shortage of anything in any aspect…by earning in morning and evening we fulfill their needs”* (Father 7). In the event that parents needed financial assistance in caring for their ill child, working together was also important. Father 5 explained, “*my brothers, my mother, my wife, and I care for the child. I mean, when the child get ill, they take money with them, and if I get short of money then they give it to me”.* Another father reached out to the *“Jamot* (people from the feudal family), *sometimes they give us a hundred or two hundred rupees; nobody gives more”* (Father 6).

Financial circumstances often dictated the treatment approach as explained by this same father:

*we do have cheaper treatment, such as for stomachache we give the child salt mixed water. When the child has constipation, then we give him or her water with sugar mixed in it; they say that the sugar water is good for constipation. We do not have anything else at home.* (Father 6)

#### Seeking home remedies prior to alternatives

The interviews were a rich source of information about the cultural practices intended to improve infant health, described in Table 2.

**Table 2:** Cultural practices

**Practice**	**Description**	**Cultural belief**
Natural Remedies	Sulemani Chaye - Tea made from boiled clove and cardamom.	Child is fed this until the milk comes in to clear the stomach.
	Satti - Strained water in which various things (from seeds to herbs and in one case cheese) have soaked. Many of the participants were unable to identify the specific ingredients that were soaked in the water. One mother indicated it was mixed with milk. Two to three drops are given to the baby once a day some mothers give it in the morning, others at night. One mother indicated it was given to the child after 40 days.	Helps the child grow up, cleans out the baby's stomach, 'normalizes' stool, prevents cramping, helps digestion, minimizes jaundice, promotes relaxation and prevent acne.
	Phenargan - a syrup fed to the baby, each night at bedtime. One mother indicated this was only given to the babies after 40 days.	Helps with allergies, stomach aches and relaxation.
	Gripe water (Nonihal) - One mother indicated it was warmed. One father indicated the ingredients were: alcohol bicarbonate, ginger, dill, fennel and chamomile mixed with water.	Treats or prevents stomach cramps, increases peristalsis, and makes the digestive tract work ‘appropriately’.
	Suji - Granulated wheat cooked in cow's milk. One mother indicated it was given after 40 days.	Helps the child to grow up healthy.
	Arq (sap) fed to the child.	Treats stomach aches, diarrhea and cramps.
	Chumber Kathi - fed to the child with warm water.	Relieves stomach aches.
	Salt water.	A cheap treatment for stomach ache.
	Sugar water.	A cheap treatment for constipation and diarrhea.
	Warm water is put on the child.	Helps a child urinate.
	Awjwain - a type of seed that is given to the mother if the child has a stomachache.	Treats stomach aches.
	Ear Drops - A few drops of warmed mustard or garlic oil (soaked with carmon seeds in one instance) are put in the ear.	Treats ear aches.
	Pan Ash - Black ash is mixed with salt and mothers milk then fed to the baby.	Treats stomach aches.
	Milk Drops - Breast milk is dropped into the eyes or nose.	Treats general infection.
Sero (Surma)	Black powder is applied to the eyebrows.	Promotes the growth of thick dark eyebrows.
	Black powder is applied in the eyes (similarly to eyeliner application).	Prevents a child's eyes from becoming weak, it keeps them clean and makes the eyes big and dark in colour.
	Black powder is applied to the umbilicus after it sheds off.	Helps to close the hole, ensures air does not enter the body and makes the umbilicus ‘go in’ sooner.
Licking as a Diagnostic Tool	The back of a baby's hand or stomach is licked.	If the skin tastes salty then the child has a stomach ache.
Wrapping the Child's Head	The child's head is oiled then wrapped and placed on a pillow (sometimes made of ash). One mother indicated that this was done for the first 40 days.	This technique is used to ensure the child's head is properly formed.
Wrapping the Child's Body	Babies are wrapped when they sleep.	Some mothers indicated this was done to prevent startling while sleeping. Others indicated that the practice ensured that the body was formed properly.
Baby Massage	Babies are massaged each morning usually with mustard oil (or coconut or olive oils).	This keeps the baby healthy and in one case 'removes the laziness' from the child so he/she will sleep calmly.
Umbilicus Treatment	Mustard oil is mixed with salt or tumeric or both and applied to the umbilical cord for a few days until it falls off.	Helps the umbilicus dry out, shed, and 'normalize'.
Parrainrr (dum)	Parrainrr - the cultural practice of a healer.	Babies are taken to a healer to stop crying, treat seizures and protect the child against ghosts.
Twin Fingers (Jarrion Ungrrion)	A cultural practice performed by a healer.	Treats stomach aches.
Knife to Protect the Baby	A knife (or some form of iron) is kept near the baby at all times.	Protects the young baby from ghosts.
Sunlight Exposure	Children are kept outside in the cold morning sun for between 5-15 minutes (although one mother indicated 3-4 hours). In some cases the babies are taken into the sun after their morning massage when they have oil on their skin.	The exposure to sunlight promotes health and treats jaundice.

Participants explained that they initially relied on home remedies and “*If the child does not get well then, we will have to take him to the doctor, in compulsion”* (Mother 6). Although the mothers strived to take care of their babies in the best way possible, some of the mothers had little or no knowledge about the ingredients used in the natural remedies. One mother explains that the satti that she feeds to her child is prepared by her elders: *“Our elders used to put (together the ingredients for satti); we don’t know (what they are)*” (Mother 1). Decisions to seek medical care were also influenced by the extent of the illness, as explained by a Grandmother 3: *“Then we will try to use some home remedies. If there is too much problem, then we will take baby to the hospital* (Grandmother 3). Another grandmother indicated that traditional remedies are sometimes chosen at night time when going to the doctor is not an option:

*The elders will say that ‘put some salt in the pan’s ash and give it to the child if he suffers through stomach ache at night’; because at that time we cannot take the child to the doctor, so we do treat it like this.* (Grandmother 1)

Alternatives to home remedies (i.e., care by doctor, care in the hospital, or medications) were not readily embraced by all given their past experience with the health care system or treatment strategies. Given what she observed when her daughter was admitted, one mother expressed, *“I feel scared at [a hospital]”* (Mother 3). Another mother explained:

*My husband forbids me. He says that ‘don’t take too much medicines’. Our first child expired after taking medicines, when he received injection. So that means he dies.* (Mother 7)

Regardless of when a family seeks medical attention for their child, they are acting in the best way they know how.

#### Sons and daughters equally important

The impact of having a son or a daughter on a mother’s status within the family in decision-making was explored. All participants indicated that “*the son and the daughter are considered equal”* and that *“the son is not given more importance*”. This importance extended to women who marry into families: *“We give more importance to our daughters-in-law because they are also somebody’s daughters and they have come for our sons. They also give us children, so why shouldn’t we give them importance?”* (Grandmother 2).

However, this perspective was not always dominant in the community: *“I had an aunt who had six daughters. She did not have any son so her husband, mother-in-law, and father-in-law used to beat her”* (Grandmother 3). The same grandmother shared that *“our men used to hate daughters previously; now they do not hate daughters, now they are considered equal”.* Grandmother 4 offered an explanation for this change in perspective: *“they came to our village so their minds changed. Here, they got education and they came to know about these worldly matters”.* When asked to reflect on the influences which led to the change in valuation, she explained: *“If a person is ill, the daughter will care for him or her”.*

The above themes describe the essence of the mothers’ experience to maintain health or improve the health outcome of her newborn which can be characterized as “loving care in silence”. Loving care necessitates acquiring knowledge and skills related to infant care including seeking home remedies when the infant in unwell. The silence embodies quiet acceptance of decisions by the extended family members, especially the paternal grandmother, regarding seeking health care and having faith in God for good health outcomes for her newborn.

## Discussion

Participants described a multigenerational approach to decision-making concerning infant care, including parents, grandparents, aunts and uncles, and sometimes the community. Parents deferred to elders when the infant was unwell, consistent with the cultural value of gaining experience and knowledge through practice. Parents learned to be caregivers as they raised their infants. When parents had successfully raised their children and overcame adversity, they then reached a status allowing involvement in decision-making and in teaching future generations. One factor which may be protective for child mortality [20] is how families work together to care for their infant; paradoxically at times, may put the infant at risk of death. This rich support network offers much help for the parents to improve health knowledge and care.

However, women were also bound by social ties, unable to act autonomously or have a voice in care decisions. This may be unsettling to mothers, raising confusion, guilt, and uncertainty about their mothering practices [21]. In a study of Pakistani women, perceived lack of social support was a risk factor for postnatal depression [22]. If mothers are unable to articulate their opinions, both their health and the health of their infant may be compromised. Conversely, fathers would do anything for their child, sometimes going against the elders, particularly when traditional approaches were not improving the health of their infant. Changes within this community will not occur when working solely with mothers, as most do not make decisions about their child’s care. Having the understanding and support of the community and all family members is pivotal to designing culturally appropriate, effective community-based interventions.

Participants were concerned with the physiological, safety, and comfort needs of the infant, although limited consideration was given to supportive caregiving (e.g., promoting infant’s self-regulation and sense of competence), and promotion of love and affection through social interactions. Participants relied on late hunger cues (i.e., crying) rather than early cues (e.g., sucking on fingers, fussiness) to determine readiness to feed [23,24]. Furthermore, both mothers and fathers expressed little knowledge of parenting skills. Caregivers’ sensitivity to and timely response to infant cues (i.e., contingent responsiveness) and the reciprocal way in which infants responds, shapes social interactions between caregiver and infant [25,26]. Caregiver-infant interactions that are less than optimal–lacking warmth, sensitivity, and responsiveness to infant cues–may result in toxic stress that can influence developmental outcomes and alter health in later life (i.e., increase vulnerability to disease) [27].

Many cultural practices require introducing non-breast milk substances into infant diets before 6 months of age. Although most traditional fluids and semi-solids ingested by babies appeared to be in very small quantities, these feedings may still displace some milk in an infant’s diet. Displacing breast milk with other substances has been associated with reduced duration of breastfeeding and more extensive supplementation [28] and places infants at higher risk for infection and diarrhea. Additionally, mothers had highlighted little understanding of cultural practices performed on their babies. Several cultural practices designed to protect a baby’s health, such as applying surma onto baby’s eyes, possibly put them at increased risk. Surma often contains harmful concentrations of lead [29]. Treating jaundice with sun exposure was a practice commonly identified by interviewees, although some research indicates concerns about the risks of skin damage from UV rays during the treatments [30]. While some cultural practices are concerning, others can benefit for the babies’ health, such as the use of breast milk to treat infections in the nose and eyes [31], and infant massage, which has no reported risks to infant health [32].

It was apparent that decisions families made for their infants was to better their health. The families grew up watching the practice of traditional remedies by known people their community, and medical interventions may be less familiar to them. The tensions forming between traditional remedies and medical treatment are evident. As future interventions are planned with communities, the deep connection with cultural practices must be respected to effectively work with families, but more importantly, to preserve the dynamic relationships that exist within the family system.

In Pakistan, *son preference* is common and women’s status within the family often rises after the birth of a son [33]. In contrast to assumptions of the researchers prior to the interviews, all participants indicated that sons and daughters are equal. This discovery of birth-gender equality is important as it reminds researchers to draw attention to their biases preventing influences of stereotyping in the analysis.

Beyond reducing neonatal mortality rates, Millennium Development Goal 4 also proposes that 90% of first-level care facilities deliver integrated management of newborn and child illness (e.g., pneumonia, malaria) to reduce under-five mortality by two thirds [3]. Affordability was a barrier for participants and limited access to such first-level care services demonstrates the fragility of sustaining and potential for regression on Millennium Development Goal 4, particularly during periods of economic and political instability. In low- and middle-income countries, income and child mortality are inversely related [7]. Similar to other South-Asian communities, women in Ibrahim Haidery were unable to negotiate decisions to seek care for themselves (e.g., antenatal services) [34] or their infants, further exemplifying that some Millennium Development Goals are not on track (i.e., Goal 3 Promote gender equality and empower women). Progress towards all Millennium Development Goals (e.g., reducing poverty) will be important to achieving the goal of reducing childhood mortality.

Women were interviewed in their own language, enabling them to tell their story without interruption. As well, SK speaks and writes Sindhi, thus able to verify the transcripts of audiotape and subsequent translation of text to English. One limitation of the study is that participants were interviewed about decisions regarding care seeking for their newborn; however, only 3 of the 10 newborns had experienced illness since birth. Most mothers (70%) were multiparous, thus experiences from their previous births framed current decisions about caring for their newborn. We did not clarify and/or expand themes, emergent ideas, or concepts with the participants in a community feedback process [16], but maintained rigor and credibility by returning to participants’ raw data (i.e., audiotape) when co-analyzing the data to prevent preconceptions from distorting the reality of participants’ experiences. The in-depth interpretation of data and rich descriptions of the participants’ demographic and sociocultural context, as well as the research context also serve to increase the transferability of the results.

## Conclusion

This study demonstrates that many cultural influences make working with families more intricate than is initially obvious. For instance, health seeking behaviors is within the purview of the primary decisions-maker and the shifting emphasis in health seeking behaviors when an infant’s health does not improve or deteriorates. Additionally, understanding family financial constraints is significant to the choices they make. If health care workers want to impact lives in this community and improve neonatal survival rates, the family context within their community is an important factor that needs to be considered in managing interventions. Health care accessibility must also be evaluated. Health care in Pakistan is a mixture of private and publically funded care, and facilities are not always easily accessible for the community members and are not always open at night [35].

## Competing interests

The authors declare that they have no competing interests

## Authors’ contributions

SP, SK, and SM were responsible for the study conception and design, as well as data collection. SP and RF independently analyzed the data. All authors contributed to the draft of the manuscript and SP integrated the various contributions and made critical revisions to the content. SK and SM made critical revisions to the paper for important intellectual content. All authors read and approved the final manuscript.

## Authors’ information

S Premji, BSc, BScN, MScN, PhD is an Associate Professor in the Faculty of Nursing, Adjunct Associate Professor in Faculty of Medicine, Department of Community Health Sciences, University of Calgary.

S Khowaja, BScN, MScN. MA (EHPID) is a Senior Instructor in the School of Nursing and the Department of Community Health Sciences, Aga Khan University.

S Meherali, BScN, MSc is a PhD candidate in the Faculty of Nursing, University of Alberta.

R Forgeron was a BScN student in the Faculty of Nursing, University of Calgary.

## Pre-publication history

The pre-publication history for this paper can be accessed here:

http://www.biomedcentral.com/1471-2393/14/232/prepub
